# Effectiveness of a multicomponent treatment versus conventional treatment in patients with fibromyalgia: Study protocol

**DOI:** 10.1097/MD.0000000000018833

**Published:** 2020-01-24

**Authors:** Felipe Araya-Quintanilla, Héctor Gutiérrez-Espinoza, María Jesús Muñoz-Yánez, Iván Cavero-Redondo, Celia Álvarez-Bueno, Vicente Martinez-Vizcaíno

**Affiliations:** a Rehabilitation and Health Research Center. CIRES, Universidad de las Américas; b Faculty of Health Sciences, Universidad SEK; c Physical Therapy Department, Clinical Hospital San Borja Arriaran, Santiago, Chile; d Universidad de Castilla-La Mancha, Health and Social Research Center, Cuenca, Spain; e Universidad Politécnica y Artística del Paraguay, Asunción, Paraguay; f Facultad de Ciencias de la Salud, Universidad Autónoma de Chile, Talca, Chile.

**Keywords:** Conventional treatment, fibromyalgia, multicomponent treatment, pain, randomized clinical trial

## Abstract

**Background:**

Fibromyalgia (FM) is a chronic pain syndrome characterized by widespread musculoskeletal pain and multiple symptoms. It is a common clinical condition whose etiology is unclear. Currently, there is no gold standard treatment for FM. Management of this condition is therefore aimed at reducing symptoms and maintaining the individual's ability to function optimally. Based on the principal symptoms and characteristics of individuals with FM, we hypothesized that the implementation of a multicomponent treatment (with physical exercise, cognitive behavioral therapy adding to a graded motor imagery program, and therapeutic neuroscience education) would be more effective than conventional treatment in women with FM. This paper describes the rationale and methods of study intended to test the effectiveness of multicomponent treatment versus conventional treatment in patients with FM.

**Method/Design:**

Fifty-six female individuals between 18 and 65 years of age, who were referred to the physical therapy department of the Rehabilitar Center in Chile, will be randomized into two treatment arms. The intervention group will receive a multicomponent treatment program for duration of 12 weeks. The control group will receive a conventional treatment for this condition for 12 weeks. The primary outcome measure will be the pain intensity score, measured by the numeric pain rating scale (NPRS), and the secondary outcomes will be the FM Impact Questionnaire (FIQ), and affective components of pain, such as catastrophizing using the Pain Catastrophizing Scale (PCS), fear of movement using the Tampa Scale Kinesiophobia (TSK), and sleep quality as measured by the Pittsburgh Sleep Quality Index (PSQI).

**Discussion:**

This paper reports the design of a randomized clinical trial aimed at assessing the effectiveness of the multicomponent treatment versus conventional treatment in women with FM.

**Trial registration:**

Brazilian registry of clinical trials UTN number U1111-1232-0862. Registered 22 April 2019.

## 1 Introduction

Fibromyalgia (FM) is a chronic pain syndrome characterized by widespread musculoskeletal pain and multiple symptoms, including fatigue, sleep disturbance, cognitive dysfunction, and psychological distress.^[1,2]^ It is one of the most common chronic pain conditions, with a worldwide prevalence of 2%, and is found in 1% to 4.9% of women and in 0% to 2.9% of men.^[3,4]^

The etiology of FM is unclear; however, central mechanisms are strongly implicated, including evidence of abnormalities in structure, function, and molecular chemistry of the central nervous system.^[5–7]^ A common finding in chronic pain syndromes is central sensitization, which is defined as an increased responsiveness of the central nervous system to a variety of stimuli (e.g., pressure, temperature, light, and medication).^[8]^ This central hyperexcitability causes hyperalgesia, allodynia, and referred pain across multiple spinal segments, and has been show in patients with FM.^[9,10]^

Management of FM is aimed at reducing symptoms and maintaining the individual's ability to function optimally,^[11,12]^ but currently there is no gold standard treatment for FM. Some guidelines and systematic reviews showed that the efficacy of pharmacological treatment was questionable and had a modest effect on patients with FM.^[13–16]^ Moreover, some evidence indicates that treatments which include multiple non-pharmacological components have beneficial effects on key FM symptoms, although effects are limited and often do not persist over time.^[17,18]^ Factors that could affect this poor response to treatment include fear of movement and catastrophization in individuals with FM.^[19–21]^

Multicomponent treatment, involving a combination of aerobic exercise and cognitive behavioral therapy, has shown some evidence as a moderate treatment of FM.^[22,23]^ However, the multifactorial component of FM and its wide variety of symptoms could interfere with treatment success,^[20,24]^ and affective components, such as poor pain coping, catastrophization and kinesiophobia, have been associated with poorer treatment outcomes in FM.^[21,25]^ Ignoring these individual differences may compromise the evaluation of treatment outcomes.

Graded Motor Imagery (GMI) and Therapy Neuroscience Education (TNE) are therapeutic tools successfully used in a number of conditions.^[26–29]^ The aim of GMI and TNE is to facilitate sensory and motor cortex reorganization, decrease pain, and improve function in patients with chronic musculoskeletal pain.^[30,31]^ Additionally, TNE is aimed at teaching patients about the neurobiology, neurophysiology, processing, and representation of pain in the central nervous system.^[32]^ Both treatments can be individually adapted and include neurophysiological adaptations, which could have benefits in the treatment of the individual needs of patients with FM.

Unfortunately, no evidence has been found regarding the combined use of these treatments in a multicomponent therapy for patients with FM. This paper reports the rationale and methods of a single-blinded, randomized controlled trial aimed at assessing the effectiveness of a multicomponent treatment involving physical exercise, cognitive behavioral therapy adding to a GMI program, and a TNE program, on pain relief, affective components, and functional improvement, as compared to conventional treatment in women FM patients over 18 years of age.

## 2 Method

### 2.1 Study design/setting

**Figure 1:**
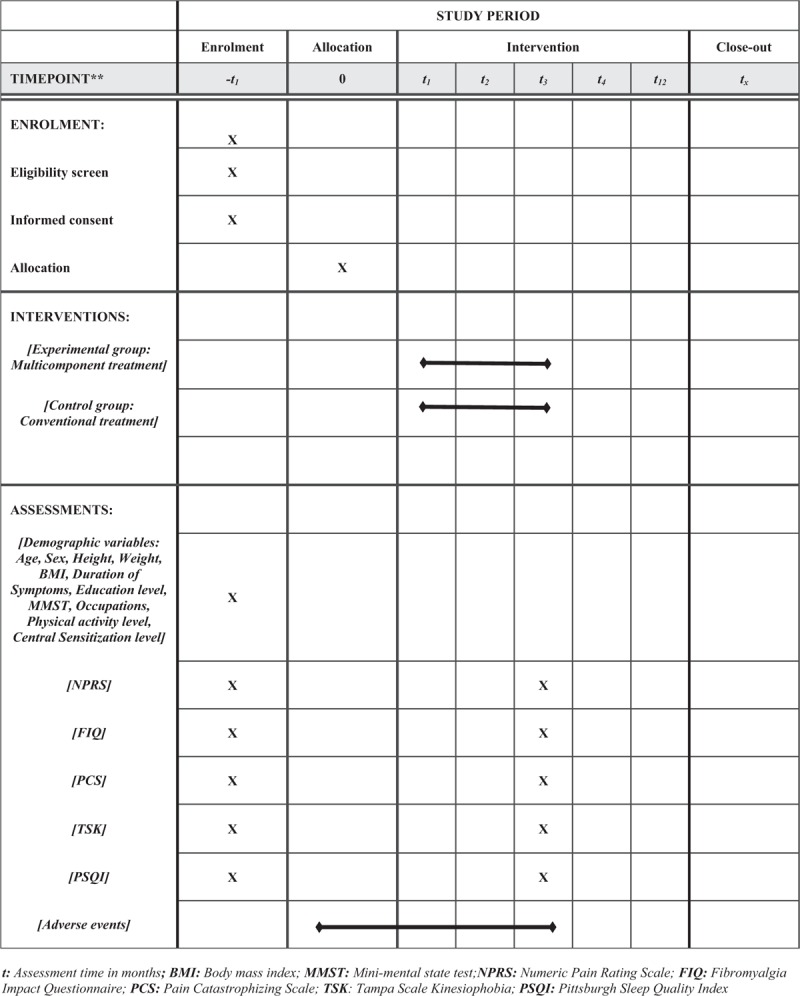
Standard protocol items: recommendations for Interventional Trials (SPIRIT) figure.

This protocol was reported based on Standard Protocol Items: Recommendations for Interventional Trials (SPIRIT) guidelines.^[33]^ This study will be a single-blinded, randomized controlled trial with two parallel groups. It will be conducted at the Physical Therapy Department of the Rehabilitar Center in Chile. The participants will be informed about the research, the procedures, the risk, and the benefits by FAQ (author of this protocol). After their agreement, participants must sign the informed consent form to be involved in the study, following the schedule as described in Figure 1.

### 2.2 Participants

A total of 56 adult females with FM, diagnosed by a rheumatologist based on the clinical history and the American College of Rheumatology criteria, will be included in this study.^[34]^ The American College of Rheumatology criteria are: general muscle pain, fatigue sensation, unrestful sleep, and cognitive dysfunction. These clinical signs have shown sensitivity and specificity values above 90% for the diagnosis of FM.^[34,35]^

### 2.3 Inclusion criteria

To participate in the study individuals must fulfil the following inclusion criteria:

1. women with FM over 18 years old attending the Physical Therapy Department of the Rehabilitar Center, with a clinical diagnosis based on the clinical criteria of the American College of Rheumatology,
2. a pain intensity above 4 out of 10 on the Numeric Pain Rating Scale (NPRS),
3. the ability to follow simple instructions, and
4. willing to accept and sign the informed consent.

### 2.4 Exclusion criteria

Conversely, patients with the following conditions will be excluded:

1. pregnancy and/or lactation,
2. chronic cancer pain,
3. metabolic disorder and/or uncontrolled comorbidities,
4. some degree of cognitive impairment, scoring < 26 points on the Mini-Mental State Test (MMST).

### 2.5 Interventions

This study will be a single-blinded, randomized controlled trial with two parallel groups. The intervention group will receive a multicomponent treatment based on physical exercise, cognitive behavioral therapy adding to a GMI program, and a TNE program. In the initial stage, TNE will be used to decrease pain, fear avoidance, and disability.^[32,36]^ The TNE component aims to educate individuals on how pain is processed by the nervous system,^[37]^ using images, examples, metaphors, and drawings, as needed, during twice-weekly sessions for 2 weeks, each session being an average of 60 min duration. To ensure a standardized TNE program, a systematic checklist will be developed. Patients will also receive a booklet summarizing the educational content of the session, including images, examples, and metaphors about chronic pain and about their clinical condition. Patients will be asked to read the TNE booklet at least once a day.

The GMI program will include three steps: laterality training, imagined movements, and mirror therapy. Laterality training is the first step in the GMI program, designed to improve the accuracy of the patient's cortical representation of his/her body. The patient trains by looking at left and right images of body parts in different positions using the Recognize application, created by the Neuro Orthopaedic Institute (NOI). The application records both accuracy and response time and allows the user to set the difficulty of the images by modifying context and background. Training is carried out for 1 h per day, in short sessions using 20 images. Laterality training will be progressed by increasing the number and difficulty of the images involved.

The second step of the GMI program will be imagined movements, aimed at preparing the patient to move. The exact instructions that will be given are: "imagine that you are involved with the body in the illustrated positions without really moving in. Imagine each position twice, and repeat the whole process three times a day.” Imagined movements will be involved with anterior flexion, abduction and rotations, depending on the body segment being treated. The patients will be provided with photographs or diagrams of the positions which are to be imagined. Imagined body movements are reported to activate the cortex in a similar way to executed movements.^[38]^

The last stage of the GMI program will be mirror therapy. The patients will be instructed to look at the mirror image of the unaffected body segment and to move that body segment in different ways. This creates the illusion that the body is moving pain–free and provides strong, positive, sensory cortical feedback that movement does not have to be painful, and may disprove cognitions of the mind, thus allowing the patient to believe pain-free movement is possible.^[39]^ Individuals are instructed to assign a body segment with the greatest symptomatology in the last 7 days.^[40]^ Patients will perform mirror therapy in two weekly sessions for 2 weeks.

An exercise program will be prescribed based on the individual's maximum heart rate, determined by polar M600^[41]^ starting just below their capacity and gradually increasing in duration and intensity. Physical activity sessions will include aerobic and stretching exercises based on the recommendations of the American Society of Sports Medicine,^[42]^ including 10 min warm-up, 40 min of effective aerobic exercise involving step, walking, and functional exercises with physiotherapy tools, and 10 min cool down including stretching exercises and a soft walk for 5 min, with deep breathing and global movements. The session will be performed twice a week for 4 weeks.

The cognitive behavioral therapy program prescribed will include group sessions of education about FM and pain perception theory, cognitive restructuring skills training, emotional stress management, and family problem resolution. All sessions will be carried out weekly for 1 h over an 8-week period.^[43]^

In the control group, all patients will receive pharmacotherapy and a conventional treatment program based on standard education by the physician. In the first session, the patient will be evaluated on comorbidities and clinical condition, and will receive health education on pain using standard images and graphic examples. Secondly, each patient will be prescribed duloxetine 60 mg, pregabalin 300 mg, milnacipran 100 mg and vitamin D 200 mg. Each dose will be evaluated and modified in relation to the requirements of each patient and taking into account the possible adverse effects.^[44,45]^ In the final session, each patient will be prescribed cyclobenzaprine 5 mg and adverse effects and the need for new medication will again be evaluated again. This will be performed once a week for 12 weeks. To monitor adherence to treatment, patients will be contacted by phone at the end of each week of treatment.

### 2.6 Outcome measures

Baseline, post-intervention outcome variables, and potential confounders will be measured in both intervention and control groups. Measurements will be taken prior to starting the treatment and at the end, at the 12th week.

### 2.7 Primary outcome measure

Pain intensity, as measured by the Numeric Pain Rating Scale (NPRS)^[46,47]^ will be the primary outcome, where 0 indicates “no pain,” and 10 indicates “worst possible pain.” At each measurement point of the study, patients of both groups will be asked to rate the average intensity of their pain over the past 7 days. This procedure has demonstrated a high degree of reliability.^[48]^

### 2.8 Secondary outcome measures

Secondary outcome measures for this study will be: the impact of FM, pain catastrophizing, fear of movement, and sleep quality.

The impact of FM is measured by the Fibromyalgia Impact Questionnaire (FIQ).^[49]^ This is a 10-item questionnaire assessing the impact of different aspects of FM on physical functioning, work status, depression, anxiety, sleep, pain, stiffness, fatigue, and well-being. The total FIQ score is calculated using a pre-determined algorithm and ranges from 0 to 100, where a higher score indicates a greater impact. The FIQ is widely used as an outcome measure for patients with FM, and its reliability and validity have been demonstrated.^[49]^

Pain catastrophizing, as measured by the Spanish version of the Pain Catastrophizing Scale (PCS),^[50,51]^ is a self-administered questionnaire that evaluates inappropriate coping strategies and catastrophic thinking about pain. The PCS uses a Likert scale of 13 items comprising of three dimensions: rumination, magnification, and hopelessness. The range of the scale is between 13 and 52 points, where low scores indicate low catastrophization and high scores indicate high catastrophization. This scale has demonstrated consistent validity and reliability.^[50]^

Fear of movement, as measured by the Tampa Scale of Kinesiophobia (TSK),^[52]^ involves a questionnaire with 17 items. Each item is scored on a 4-point Likert-type scale that ranges from strongly agree (1) to strongly disagree (4). Total scores for the questionnaire range from 17 to 68 points, where higher scores indicate more fear of movement and/or (re)injury. This scale has demonstrated consistent validity and reliability.^[53]^

The quality of sleep will be evaluated with the Pittsburgh Sleep Quality Index (PSQI), which corresponds to a questionnaire of 24 items aimed at assessing aspects related to the quality, subjective estimation, latency, frequency, and severity of problems of sleep. The maximum score is 21 points, and its reliability and validity have been previously demonstrated.^[54,55]^

### 2.9 Potential confounders

#### 2.9.1 Clinical variables

The physical activity level will be assessed by the Global Physical Activity Questionnaire (GPAQ).^[56]^ This questionnaire was originally designed by the World Health Organization to be interviewer-administered in assessing physical activity. The questionnaire comprises of 16 items that quantify the participant's physical activity level within a normal active week in order to estimate the total weekly volume of moderate-to-vigorous physical activity. It includes three domains: work, transportation, and recreational activities. This questionnaire has demonstrated consistent validity and reliability.^[57]^

Age, sex, duration of symptoms (months), and central sensitization in the last three months will be evaluated.

Anthropometry and body composition: weight will be measured with the patient barefoot and in light clothing. Height will be measured using a wall stadiometer, with the patient barefoot and upright, and with the sagittal midline touching the back board. Body mass index (BMI) will be calculated as weight, in kg, divided by the square of the height, in meters (kg/m^2^).

#### 2.9.2 Socioeconomic status

Education level will be classified as primary education (functionally illiterate, without any studies, or those not completed primary education), middle education (primary education, high school/secondary education, or baccalaureate) and university education (college or PhD degree).

The cognitive status of all patients will be evaluated using the MMST.^[58]^ This is the most commonly used test for standardized cognitive assessment in the clinical setting.^[59]^ Although the scores of patients with mild-to-moderate cognitive impairment could be influenced by a number of sociodemographic variables (such as age and educational level),^[60–62]^ we established the cut-off at 26/27 to ensure that patients could follow simple commands and therapeutic indications during treatment.

### 2.10 Sample size calculation

Sample size for this trial is based on an expected mean difference between groups of 2 points of the NPRS, which is the minimum clinically important difference.^[63]^ The mean assumed for the calculation was 5.95, with a standard deviation (SD) of 2.06 points, based on results of other randomized clinical trials.^[64]^ To detect this difference between the control and intervention groups, with a value of α = 0.05 (probability of committing a type I error) and a statistical power of 90%, a minimum of 22 patients per group is needed. This minimal sample size estimate has been increased by 20%, taking into consideration the potential dropouts, giving a total of 28 patients for each group. Sample Size was performed using the Stata SE software, version 15 (StataCorp, College Station, TX).

### 2.11 Recruitment

Recruitment of the participants begun in April 2019 and is expected to finish in December 2019. Information on the study goals and procedures will be verbally provided. The participants will also be invited to raise questions or doubts on any aspect of the study. Data confidentiality guarantees will be provided to participants by the principal investigator. Written consent will be obtained from all participants before registration, and participants may withdraw from the trial at any point in time without penalties. The written consent form includes information regarding the background and purpose of the study, therapeutic interventions, outcomes, and the expected benefits and drawbacks.

### 2.12 Randomization and blinding

**Figure 2:**
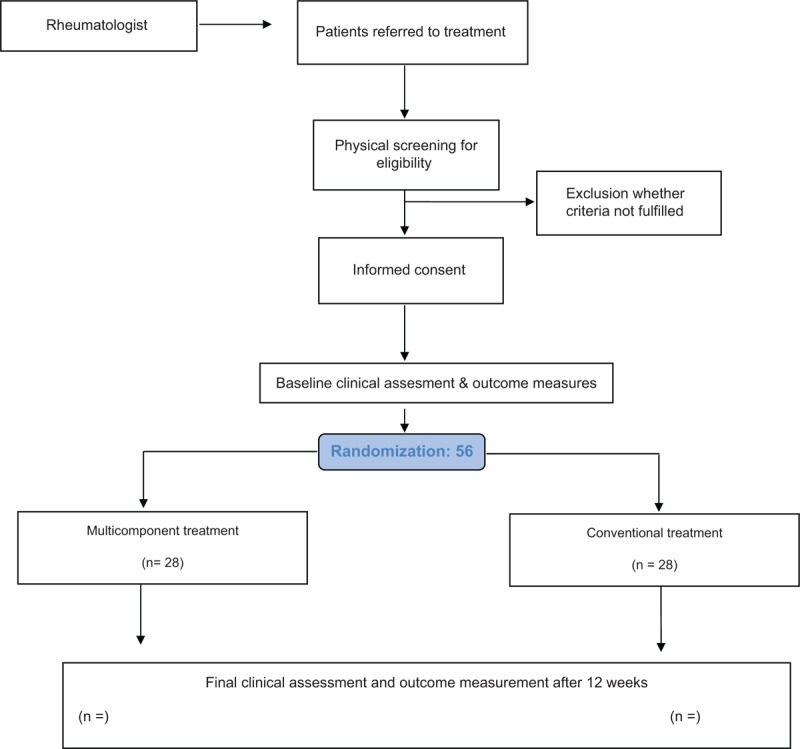
Flow diagram of patients through phases of clinical study.

Participants will be allocated to each group in a random manner through a sequence of numbers generated by computer program before the selection process begins. The group assigned to each patient will be kept in a sealed envelope, with the objective of concealing the assignment from the researcher (Fig. 2). Given the nature of the therapeutic interventions studied, physiotherapists and patient blinding will not be possible. However, the evaluator and the statistician will not know to which group each evaluated subject belongs.

### 2.13 Data management

Information obtained on the measurements of each participant will be recorded on a paper print-out. The information will then be hand-written on a paper document case report form and entered into an Excel file for future statistical analysis. In compliance with the Personal Information Protection Act, the names of all participants will not be disclosed, and a unique identifier number, given during the trial, will be used to identify participants. All participants will be informed that the clinical data obtained in the trial will be stored in a computer and will be handled with confidentiality. The participants’ written consent will be stored by the principal investigator.

### 2.14 Statistical analysis

The continuous variables will be presented as mean and standard deviation, and the categorical variables as number and percentage. To determine whether parametrical statistical tests are appropriate for the analysis of the data, the fitting to normal distribution will be evaluated using both statistical (Shapiro–Wilk test) and graphical (normal probability plot) methods. To examine baseline differences, a Student t-test will be used with interval variables, and Chi-squared with nominal ones. Analysis of covariance (ANCOVA) will be used for differences between groups at twelve weeks in primary and secondary outcomes using continuous scales, with adjustment for baseline levels of outcomes, using group differences in the mean change from baseline to twelve weeks as dependent variable. Finally, in the event of possible losses or dropouts, a statistical analysis will be carried out by protocol and intention to treat.

Data will be processed independently by two researchers, and inconsistencies will be detected using the VALIDATE command of the Epi Info (CDC) software. After checking for true outliers and extreme values, data will be winsorized using results below the 1st percentile and above the 99th percentile of the distribution of variables. Prior to analyzing the data, we will check for missing data and consider applying imputation methods using chained equations.

### 2.15 Harms

Patients from both groups will have a logbook available during each session, in order to collect, assess, report, and manage the potential adverse effects of the interventions that will be performed in the study. According to the informed consent, patients who show an increase in symptoms after 48 h of the session will be immediately evaluated by an rheumatologist.

### 2.16 Ethics

The study will be conducted under the Declaration of Helsinki principles,^[65]^ as well as the norms of good clinical practice. The study protocol has been approved by the Ethical Committee of the East Metropolitan Health Service of Chile, with the reference number 16042019. This research was registered in the Brazilian registry of clinical trials with the number U1111-1232-0862.

## 3 Discussion

The aim of this study is to describe the rationale and methods of a randomized clinical trial intended to test the effectiveness of a multicomponent treatment in FM female patients. The intervention group will receive a multicomponent treatment based on physical exercise, cognitive behavioral therapy adding to a GMI program, and a TNE program. The control group will receive a conventional treatment based on standard education and pharmacotherapy.

Some evidence has studied the effectiveness of multicomponent treatment in FM, based on aerobic exercise and cognitive behavioral therapy only.^[17,66,67]^ Although these treatments have beneficial affects on FM symptoms, these are limited and often do not persist for a long time.^[17,18]^ A recent overview of guidelines^[67]^ showed that a multicomponent treatment has inconsistent results in the management of FM, making it difficult to establish which type of exercise or specific therapy is more clinically effective in FM patients, and suggesting the needed for further studies.^[25,68,69]^

Based on available evidence, FM is a disease characterized by chronic pain and central sensibilization dysfunction,^[8,70]^ with individual variability in other symptomatology, and poor response to treatment.^[19]^ Several studies showed cortical reorganization in patients with FM,^[7,71,72]^ including changes in areas involved in pain processing and affective pain components, such as amygdala, sensorimotor cortex, insula, and morphometric changes and functional activity in cingulate cortex and mesolimbic area.^[7]^ These findings must be considered in the management of FM to improve benefits and obtain better response to treatment. GMI and TNE are therapeutic tools which have been successfully used in a number of conditions with suspected central sensibilization.^[26–29]^ The mechanism of GMI and TNE has been studied. First, laterality training improves the accuracy of the cortical representation of the body,^[30]^ movement imagery may activate the motor cortex and premotor cortex in a similar way to executed movements,^[73]^ and mirror therapy improves the activation in supplementary motor area, pre-motor cortex.^[74]^ Secondly, TNE reduces the perception and threat of pain, decreasing activation of sympathetic and motor protection systems,^[75,76]^ and, in addition, decreases the hyperexcitabilty in brain areas related with pain processing.^[77]^

Physical exercise has been defined as physical activity that is planned, structured, and repetitive, with the goal of maintaining or improving physical fitness; that is, cardiorespiratory endurance, muscular strength and flexibility.^[78]^ Studies have demonstrated that women with FM are less physically active compared with healthy women.^[79]^

To strengthen the reliability of the results, important methodological factors have been considered. Participants will be randomly assigned to both groups through a hidden allocation sequence. Furthermore, the sample size has been adjusted to take into consideration the possibility of losses or dropouts, increasing the number of patients recruited by 20% and, in the event of losses or withdrawals, a statistical analysis will be carried out by protocol and intention to treat. To minimize measurement bias, all evaluations will be performed by a trained physiotherapist, external to the research team, who will remain blinded in relation to the treatment groups, and the statistician will also remain blinded to the group assignment. The outcome measures are suitable and frequently used in clinical practice, as well as having a good level of validity and reliability.

Finally, it should be noted that our study has some limitations. The absence of a follow–up after the trial finishes does not allow us to establish the effectiveness of the therapeutic interventions in the long-term. Blinding of the patients and the physiotherapists involved in the study will not be achievable given the nature of the interventions being studied.

To the best of our knowledge, this is the first randomized clinical trial that aims to study the effectiveness of a multicomponent treatment for patients with FM, based on physical exercise, cognitive behavioral therapy adding to a GMI program, and TNE. The results of this study will provide evidence to the controversial body of knowledge relating to the effectiveness of the different modalities of multicomponent treatment in patients with FM.

## Acknowledgments

The investigators would like to thank Mrs. MD Juan Ignacio Gonzalez for her administrative support at our investigation.

## Author contributions

**Conceptualization:** Felipe Araya-Quintanilla, Héctor Gutierrez-Espinoza, María Jesús Muñoz-Yánez, Celia Álvarez-Bueno, Iván Cavero-Redondo.

**Data curation:** Celia Álvarez-Bueno.

**Formal analysis:** María Jesús Muñoz-Yánez, Celia Álvarez-Bueno, Iván Cavero-Redondo, Vicente Martinez-Vizcaíno.

**Investigation:** Felipe-Araya Quintanilla, Héctor Gutiérrez-Espinoza, María Jesús Muñoz-Yánez.

**Methodology:** Felipe-Araya-Quintanilla, Héctor Gutiérrez-Espinoza, María Jesús Muñoz-Yánez.

**Project administration:** Felipe Araya-Quintanilla, María Jesús Muñoz-Yánez, Celia Álvarez-Bueno.

**Supervision:** Hector Gutierrez-Espinoza, María Jesús Muñoz-Yánez, Iván Cavero-Redondo.

**Validation:** Iván Cavero-Redondo, Vicente Martinez-Vizcaíno

**Visualization:** María Jesús Muñoz-Yánez

**Writing – original draft**: Felipe Araya-Quintanilla, Héctor Gutiérrez-Espinoza, Celia Álvarez-Bueno, Iván Cavero-Redondo, Vicente Martinez-Vizcaíno.

Iván Cavero-Redondo orcid: 0000-0003-2617-0430.
